# Supplementary material for: Modeling glioblastoma heterogeneity as a dynamic network of cell states
Source: Mol Syst Biol. 2021 Sep 16;17(9):e10105. doi: 10.15252/msb.202010105 (PMC8444284; doi:10.15252/msb.202010105)
Supplement: Supplementary file 5 — Source Data for Figure 3 [file MSB-17-e10105-s001.zip › Figure3A_sourcedata/GSEA_3065/hallmarks_state5.GseaPreranked.1623416302988/HALLMARK_APICAL_JUNCTION.html]

Details for gene set HALLMARK\_APICAL\_JUNCTION[GSEA]

|  || Dataset | state5 |
| Phenotype | NoPhenotypeAvailable |
| Upregulated in class | na\_neg |
| GeneSet | HALLMARK\_APICAL\_JUNCTION |
| Enrichment Score (ES) | -0.28287628 |
| Normalized Enrichment Score (NES) | -0.98881215 |
| Nominal p-value | 0.49750623 |
| FDR q-value | 0.7714348 |
| FWER p-Value | 1.0 |
Table: GSEA Results Summary

  

Fig 1: Enrichment plot: HALLMARK\_APICAL\_JUNCTION      
 Profile of the Running ES Score & Positions of GeneSet Members on the Rank Ordered List

  

| PROBE | GENE SYMBOL | GENE\_TITLE | RANK IN GENE LIST | RANK METRIC SCORE | RUNNING ES | CORE ENRICHMENT || 1 | SGCE |  |  | 76 | 0.565 | 0.0177 | No |
| 2 | ITGB1 |  |  | 157 | 0.477 | 0.0309 | No |
| 3 | VCAN |  |  | 191 | 0.444 | 0.0478 | No |
| 4 | JAM3 |  |  | 276 | 0.395 | 0.0568 | No |
| 5 | VCL |  |  | 400 | 0.342 | 0.0588 | No |
| 6 | ADAM9 |  |  | 421 | 0.334 | 0.0721 | No |
| 7 | CD276 |  |  | 523 | 0.305 | 0.0749 | No |
| 8 | THY1 |  |  | 546 | 0.299 | 0.0863 | No |
| 9 | EXOC4 |  |  | 592 | 0.287 | 0.0946 | No |
| 10 | CDH11 |  |  | 630 | 0.279 | 0.1034 | No |
| 11 | MPZL1 |  |  | 666 | 0.272 | 0.1121 | No |
| 12 | NECTIN3 |  |  | 682 | 0.268 | 0.1229 | No |
| 13 | SDC3 |  |  | 728 | 0.255 | 0.1297 | No |
| 14 | CADM2 |  |  | 739 | 0.253 | 0.1403 | No |
| 15 | AMH |  |  | 767 | 0.248 | 0.1488 | No |
| 16 | DLG1 |  |  | 924 | 0.221 | 0.1415 | No |
| 17 | PKD1 |  |  | 982 | 0.213 | 0.1450 | No |
| 18 | AKT2 |  |  | 1129 | 0.195 | 0.1376 | No |
| 19 | CDH4 |  |  | 1160 | 0.190 | 0.1431 | No |
| 20 | TGFBI |  |  | 1177 | 0.188 | 0.1500 | No |
| 21 | COL16A1 |  |  | 1291 | 0.173 | 0.1453 | No |
| 22 | ADAM15 |  |  | 1307 | 0.170 | 0.1515 | No |
| 23 | NECTIN2 |  |  | 1403 | 0.161 | 0.1483 | No |
| 24 | ITGA3 |  |  | 1480 | 0.155 | 0.1469 | No |
| 25 | EGFR |  |  | 1547 | 0.147 | 0.1463 | No |
| 26 | BMP1 |  |  | 1550 | 0.147 | 0.1530 | No |
| 27 | PIK3R3 |  |  | 1584 | 0.142 | 0.1559 | No |
| 28 | ADAM23 |  |  | 1586 | 0.142 | 0.1624 | No |
| 29 | ITGB4 |  |  | 1681 | 0.135 | 0.1580 | No |
| 30 | FBN1 |  |  | 1756 | 0.129 | 0.1557 | No |
| 31 | TAOK2 |  |  | 1760 | 0.129 | 0.1614 | No |
| 32 | KCNH2 |  |  | 1767 | 0.129 | 0.1667 | No |
| 33 | CX3CL1 |  |  | 1776 | 0.128 | 0.1718 | No |
| 34 | MMP2 |  |  | 1824 | 0.125 | 0.1723 | No |
| 35 | CERCAM |  |  | 1837 | 0.124 | 0.1767 | No |
| 36 | CD99 |  |  | 1945 | 0.116 | 0.1700 | No |
| 37 | NLGN3 |  |  | 1947 | 0.115 | 0.1753 | No |
| 38 | NFASC |  |  | 1999 | 0.112 | 0.1747 | No |
| 39 | NF1 |  |  | 2105 | 0.105 | 0.1678 | No |
| 40 | SYMPK |  |  | 2177 | 0.100 | 0.1644 | No |
| 41 | PTEN |  |  | 2191 | 0.099 | 0.1676 | No |
| 42 | TSPAN4 |  |  | 2226 | 0.096 | 0.1682 | No |
| 43 | BAIAP2 |  |  | 2289 | 0.092 | 0.1655 | No |
| 44 | B4GALT1 |  |  | 2347 | 0.089 | 0.1632 | No |
| 45 | PLCG1 |  |  | 2435 | 0.083 | 0.1573 | No |
| 46 | MDK |  |  | 2458 | 0.082 | 0.1586 | No |
| 47 | SPEG |  |  | 2465 | 0.082 | 0.1617 | No |
| 48 | TSC1 |  |  | 2593 | 0.074 | 0.1509 | No |
| 49 | EPB41L2 |  |  | 2855 | 0.060 | 0.1242 | No |
| 50 | PTK2 |  |  | 2987 | 0.052 | 0.1119 | No |
| 51 | INSIG1 |  |  | 3055 | 0.049 | 0.1066 | No |
| 52 | NLGN2 |  |  | 3127 | 0.046 | 0.1008 | No |
| 53 | LAYN |  |  | 3130 | 0.045 | 0.1027 | No |
| 54 | MAP4K2 |  |  | 3132 | 0.045 | 0.1047 | No |
| 55 | VAV2 |  |  | 3172 | 0.043 | 0.1022 | No |
| 56 | TMEM8B |  |  | 3294 | 0.037 | 0.0903 | No |
| 57 | PIK3CB |  |  | 3409 | 0.031 | 0.0789 | No |
| 58 | EVL |  |  | 3508 | 0.027 | 0.0691 | No |
| 59 | MAPK14 |  |  | 3545 | 0.025 | 0.0662 | No |
| 60 | MAPK11 |  |  | 3840 | 0.014 | 0.0338 | No |
| 61 | PBX2 |  |  | 3944 | 0.009 | 0.0226 | No |
| 62 | INPPL1 |  |  | 3975 | 0.008 | 0.0196 | No |
| 63 | TIAL1 |  |  | 4017 | 0.006 | 0.0152 | No |
| 64 | SIRPA |  |  | 4174 | -0.000 | -0.0023 | No |
| 65 | MVD |  |  | 4194 | -0.001 | -0.0044 | No |
| 66 | CNTN1 |  |  | 4200 | -0.001 | -0.0049 | No |
| 67 | CTNND1 |  |  | 4246 | -0.003 | -0.0098 | No |
| 68 | MPP5 |  |  | 4625 | -0.019 | -0.0516 | No |
| 69 | RASA1 |  |  | 4642 | -0.020 | -0.0524 | No |
| 70 | GNAI1 |  |  | 4664 | -0.021 | -0.0538 | No |
| 71 | ACTN4 |  |  | 4829 | -0.027 | -0.0711 | No |
| 72 | DHX16 |  |  | 4899 | -0.029 | -0.0775 | No |
| 73 | MAP3K20 |  |  | 4920 | -0.030 | -0.0784 | No |
| 74 | IRS1 |  |  | 5518 | -0.054 | -0.1432 | No |
| 75 | SHC1 |  |  | 5833 | -0.067 | -0.1754 | No |
| 76 | ARHGEF6 |  |  | 5891 | -0.069 | -0.1786 | No |
| 77 | SKAP2 |  |  | 5953 | -0.072 | -0.1822 | No |
| 78 | MYH10 |  |  | 5961 | -0.072 | -0.1796 | No |
| 79 | NEXN |  |  | 6052 | -0.077 | -0.1862 | No |
| 80 | IKBKG |  |  | 6141 | -0.082 | -0.1923 | No |
| 81 | AKT3 |  |  | 6191 | -0.084 | -0.1939 | No |
| 82 | PARD6G |  |  | 6291 | -0.090 | -0.2009 | No |
| 83 | SORBS3 |  |  | 6338 | -0.092 | -0.2018 | No |
| 84 | LIMA1 |  |  | 6339 | -0.092 | -0.1975 | No |
| 85 | PARVA |  |  | 6484 | -0.100 | -0.2090 | No |
| 86 | TJP1 |  |  | 6500 | -0.101 | -0.2060 | No |
| 87 | STX4 |  |  | 6687 | -0.113 | -0.2217 | No |
| 88 | VASP |  |  | 6754 | -0.118 | -0.2237 | No |
| 89 | SHROOM2 |  |  | 6849 | -0.125 | -0.2285 | No |
| 90 | CRAT |  |  | 6922 | -0.131 | -0.2305 | No |
| 91 | HADH |  |  | 7129 | -0.148 | -0.2468 | No |
| 92 | MYH9 |  |  | 7248 | -0.161 | -0.2526 | No |
| 93 | MSN |  |  | 7491 | -0.190 | -0.2711 | No |
| 94 | RSU1 |  |  | 7513 | -0.192 | -0.2645 | No |
| 95 | ACTN1 |  |  | 7520 | -0.193 | -0.2562 | No |
| 96 | GTF2F1 |  |  | 7581 | -0.201 | -0.2536 | No |
| 97 | NF2 |  |  | 7842 | -0.242 | -0.2716 | Yes |
| 98 | TUBG1 |  |  | 7846 | -0.243 | -0.2607 | Yes |
| 99 | RRAS |  |  | 8040 | -0.283 | -0.2692 | Yes |
| 100 | CTNNA1 |  |  | 8047 | -0.284 | -0.2567 | Yes |
| 101 | CNN2 |  |  | 8107 | -0.298 | -0.2495 | Yes |
| 102 | ZYX |  |  | 8196 | -0.326 | -0.2442 | Yes |
| 103 | WASL |  |  | 8219 | -0.331 | -0.2313 | Yes |
| 104 | CAP1 |  |  | 8335 | -0.363 | -0.2274 | Yes |
| 105 | GAMT |  |  | 8449 | -0.407 | -0.2212 | Yes |
| 106 | GNAI2 |  |  | 8540 | -0.452 | -0.2103 | Yes |
| 107 | HRAS |  |  | 8633 | -0.514 | -0.1968 | Yes |
| 108 | FSCN1 |  |  | 8710 | -0.573 | -0.1787 | Yes |
| 109 | ARPC2 |  |  | 8725 | -0.586 | -0.1530 | Yes |
| 110 | YWHAH |  |  | 8726 | -0.587 | -0.1257 | Yes |
| 111 | ACTG1 |  |  | 8859 | -0.718 | -0.1071 | Yes |
| 112 | MYL12B |  |  | 8891 | -0.760 | -0.0753 | Yes |
| 113 | ACTB |  |  | 8912 | -0.793 | -0.0406 | Yes |
| 114 | PFN1 |  |  | 8977 | -1.045 | 0.0008 | Yes |
Table: GSEA details [plain text format]

  

Fig 2: HALLMARK\_APICAL\_JUNCTION: Random ES distribution      
 Gene set null distribution of ES for **HALLMARK\_APICAL\_JUNCTION**
